# Maneuvering the care puzzle: Experiences of participation in care by frail older persons with significant care needs living at home

**DOI:** 10.1080/17482631.2021.1937896

**Published:** 2021-07-15

**Authors:** Anna Olaison, Elisabet Cedersund, Jan Marcusson, Eva Valtersson, Annette Sverker

**Affiliations:** a Department of Culture and Society – Division Social Work, Linköping University, Linköping, Sweden; b Department of Culture and Society – Division of Ageing and Social Change, Linköping University, Norrköping, Sweden; c Department of Health, Medicine and Caring Sciences, Linköping University, Linköping, Sweden; d Department of Activity and Health and Department of Health, Medicine and Caring Sciences, Linköping University, Linköping, Sweden; e Department of Rehabilitation Medicine and Department of Health, Medicine and Caring Sciences, Linköping University, Linköping, Sweden

**Keywords:** Older people, significant care needs, health care, primary care, participation, caregiving, relationship-based care, qualitative research

## Abstract

**Purpose:**

Despite evidence that older persons want to be involved in care, little is known about how frail older people with significant care needs living at home experience participation in care provided by different stakeholders. This study investigates the experiences of participation in care by older people following their involvement in an intervention of a health care model called Focused Primary care (FPC).

**Methods:**

Individual semi-structured interviews were conducted with 20 older persons in five municipalities in Sweden.

**Results:**

The results show that older persons highlighted opportunities and limitations for participation on a personal level i.e., conditions for being involved in direct care and in relation to independence. Experiences of participation on organizational levels were reported to a lesser degree. This included being able to understand the organizational system underpinning care. The relational dimensions of caregiving were emphasized by the older persons as the most central aspects of caregiving in relation to participation.

**Conclusions:**

Primary care should involve older persons more directly in planning and execution of care on all levels. An ongoing connection with one specialized elderly team and a coordinating person in Primary care who safeguards relationships is important for providing participation in care for frail older persons with significant care needs living at home.

## Introduction

The health care needs of frail older people are often extensive, multilayered, and change over time. In current policies in Sweden, like other Western countries, there is an explicit aim to reduce hospital-based care and institutional care to encourage a larger proportion of frail older people with significant care needs to live at home and to be active participants in the care and services they receive (G. Harvey et al., 2018; Lindblad et al., 2018). This is often specified as a general aim that benefits the ageing population as well as ageing societies (Lawrence, 2017). However, to achieve older persons’ participation in care requires collaboration between different stakeholders of care (Breitholtz et al., 2013; Hansson et al., 2018) and active involvement from the frail older persons with significant care needs who are at high risk of hospital admission (Fjordside & Morville, 2016; Wiles et al., 2012). Previous research has problematized interprofessional collaboratio between different health care-based and municipal-based organizations regarding the coordination of care for older people (Larsen et al., 2017; Sutcliffe et al., 2008), where health care professionals find shortcomings in regard to participation in care decisions by older persons (Foster et al., 2017; Meyer et al., 2018). However there are fewer studies focusing directly on how frail older people with significant care needs living at home themselves experience care and how they see their own opportunities to participate in the care provided.

The reported study is a sub- study with focus on interviewing frail older persons about their experiences of participation in care received through a new health care intervention. The focus of the research is directed towards how the individuals describe and value participation in this care context.

The intervention called Focused Primary Care (FPC) was carried out in Sweden during 2017–2019 (Marcusson, et al., 2019, Nord, et al., 2020). The intervention (FPC) included a tool for prediction primarily aimed at identifying individuals in great need of hospital care in the near future. Using a prediction of those older persons with great needs in order to meet those needs with individualized and differentiated care in a stepwise mode, the focus in the overall intervention study was to investigate to what extent this model is of higher quality and efficiency regarding resources for health care in comparison to the usual care given today. Together with the prediction a comprehensive geriatric assessments of the older persons’ medical status and their need of health and social care was carried out. The intention with this geriatric assessment was to get a multidimensional assessment of the health of the older persons, and with a starting point from this assessment create an interactive care plan based on the individual’s needs and preferences by a spezialized elder care team in the context of primary care. The focus of the reported sub-study has been on one part of the intervention, namely on the participating older persons and their experiences of involvement in care that they have received through the intervention.

## Research on older persons living at home and their experiences of care

Older people are often positioned as passive recipients of care by professionals in health care settings (Angus & Reeve, 2006; Lawrence, 2017). Studies however highlight that older persons want to be involved in their own care (Bastiaens et al., 2007; Bonifas et al., 2014) but they can have ambivalent attitudes towards their experience of receiving care as it has both positive and negative aspects (Sundström et al., 2018). Positive aspects include possibilities to be engaged in decisions regarding care, where care includes reciprocity, respect, safety and security (De São José et al., 2016). Negative aspects include difficulties in asking for care, disengagement in decisions on care, and multiple forms of loss in relation to receiving care for the older person, for example, loss of autonomy and confidence in performing everyday tasks (c.f. Åberg et al. 2020; Breitholtz et al., 2013). However, for frail older people significant care needs receiving care does not per se inevitably imply negative or positive experiences (De São José et al., 2016). It is instead the relational dimensions of caregiving that are highlighted as the most central aspects (c.f. G. Harvey et al., 2018; Soodeen et al., 2007), including the concrete performance of care provision as found mainly in the attitudes and behaviours of professionals (Janlöv et al., 2006; Larsen et al., 2017). The older persons themselves also refer to tensions in relation to attitudes towards seeking help and the quality of communication and information exchange (D. Harvey et al., 2017; Martinsson et al., 2012). According to several studies, this is a result of the embeddedness of care relationships in Western cultures that are characterized by the high value given to independence and an active ageing process (Lawrence, 2017; Tanner, 2010), and is also because the organizational systems that deliver care have a hierarchical structure that is characterized by bureaucracy and formality (De São José et al., 2016).

A common theme in the literature on older people’s experiences of receiving care at home is that participation in care requires both participation from the older persons involved (Fang et al., 2016; De São José et al., 2016) and cooperation in the care planning processes where health care professionals need to be inclusive (e.g., Price et al., 2018; Sundström et al., 2018). According to Fjordside and Morville (2016) older people’s participation in the planning and execution of their care is vital but the organization of care between different stakeholders often restricts older people’s scope for autonomy. Several studies have found that older people experience that they have minimal or no influence as regards modifying their care planning and the help and services they receive (Breitholtz et al., 2013; Doyle et al., 2012). Older people describe a negotiation between the assigned services as a struggle between receiving help and gaining control over their lives. They feel that they can influence the arrangements for the services but not the scope or content of the care (Price et al., 2018; Sixsmith et al., 2014). In summary, research has shown that older people have ambivalent attitudes towards their experiences of receiving care, and generally they have a lack of control over their own possibilities for participation. There is however a knowledge gap in the literature regarding frail older persons living at home with significant care needs that concerns what participation in care means for them and what this way of receiving care might imply for their well-being. It is therefore important to more effectively target and identify how care for frail older people with significant care needs should be performed by investigating how older people living at home experience their participation in care. This is also an essential question to address within research on health and primary care.

## Aim and research questions

The aim of the sub-study was to explore the experiences of participation in care by frail older people with significant care needs following their recent involvement in an intervention using a new health care model called Focused Primary care (FPC). The focus of the research is directed towards how the individuals describe and value participation in this care context. The following research questions are in focus.

*What opportunities and limitations do frail older persons with significant care needs see for participation in the care provided?*

*How do the older persons see opportunities and/or limitations for participation in relation to individual solutions regarding care and services delivered?*

## Setting

In Sweden the basic health and medical care is generally referred to as primary care. Most often primary care is provided in multidisciplinary teams, with at least a GP and a nurse, but often also with social workers, psychologists and physiotherapists working at the Primary care centre. The general medical practitioners (GP) offers medical examinations care and treatment for the most common medical conditions. However, if necessary the general practitioner can refer patients to other medical specialists (National Board of Health and Welfare, 2019). A revision of the Swedish elderly care has identified a need for developing primary care, which is characterized by a holistic view on health of older people. Primary care has also been suggested to take on a coordinating role between different actors in the welfare system in which community care and acute care for older people is included (SKR, 2018).

Community care is often a complement to primary care for patients who live at home. Municipal health and medical care refers to the health and medical care for which the municipalities are responsible. The municipalities have a responsibility for health and medical care in special forms of housing, day to-day activities and home care in ordinary housing. Municipal health and medical services is most commonly given in ordinary housing and a certain part is given in special housing. The majority of the patients are 65 years or older (National Board of Health and Welfare, 2019). The older persons interviewed in our sub- study all had opportunities to access care and services from Primary care, community care and acute care.

## Description of the Focused Primary Care (FPC) intervention

**Figure 1.**
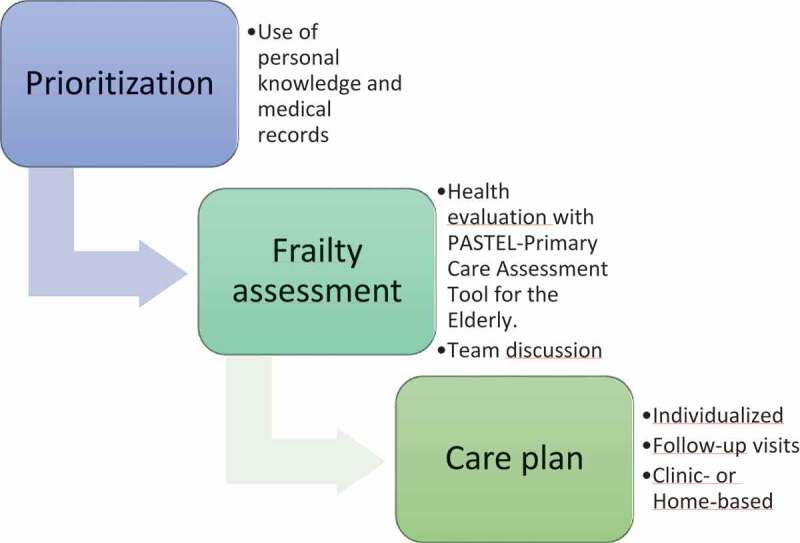
The care chain for patients included in the Focused Primary Care (FPC) intervention

The intervention project (FPC), a tool for prediction, has been developed, to identify individuals in great need of hospital care in the future. The primary aim of the intervention project Focused Primary Care (FPC) was to investigate the extent to which a differentiated and directed primary care intervention provided to a digitally predicted risk population of older persons results in care that is more efficient and of higher quality than that provided to a control group receiving standard care. Focusing on the question *Can the prediction of frail older individuals at high risk of hospital care, combined with proactive healthcare, lead to a decrease in healthcare use and costs*. In a large multi-centre study over two years (2017–2019) in primary care centres of the Region Östergötland (RÖ), regular care with the enhanced primary care (includes both health care and community care) were compared. The FPC project also includes several sub-studies of the care model. These sub-studies concern the health care system from the perspectives of specific health care professionals (primary care nurses, physicians, para-medical staff) to management levels and politicians that may influence the implementation of the present and future care models. Finally, the project also investigates the older persons, and their social networks. The reported sub study concerns the older persons experiences of participation in care (For a more detailed description of the intervention, see Authors, 2019). Figure 1 below describes the care process in the Focused Primary Care intervention.

The primary care team in the intervention project is represented by the general practitioner (GP) responsible for the patient, a registered nurse (RN) dedicated to care for older persons (and when needed a physiotherapist and an occupational therapist). The personalized intervention consists of a complete check-up/follow-up and intervention addressing medical, psychiatric, functional and social aspects of the client in a stepwise resource differentiated way according to needs based on the clinical judgement of the team. First, individuals were selected for the intervention through the use of a prediction model. The algorithm calculates a risk score for hospitalization based on age, health care utilization and selected diagnoses (Marcusson et al., 2020). The individuals with the highest risk score of the total population aged 75 years and more were selected, approximately 10%. (Step 1 Prioritization Figure 1). Thereafter, the chosen individuals were approached by the primary care team, which evaluated the older person’s social and medical condition using an evaluation form called PASTEL- Primary Care Assessment Tool for the Elderly (Step 2 Frailty Assessment Figure 1). The evaluation form was based on the holistic approach of Comprehensive Geriatric Assessment, which can be regarded as a combination of diagnostic and therapeutic processes where problems are identified and managed.[1] By using the evaluation form, the primary care team established an interactive care plan for individuals in need. (Step 3 Care plan Figure 1). The evaluation process used communication over the phone as well as visits to the primary care centre, depending on the priority of the client’s needs. Examples of common actions/measures were evaluation of medication, initiation of home care, diet treatment, advice on physical activity, and counselling for loneliness and isolation. The formation of the “elderly team”, with dedicated nurses who function as personal nurses for the frail individuals, is the key component of the intervention, together with the standardized PASTEL evaluation based on comprehensive geriatric assessment.

## Methods

### Design

The reported sub-study was of explorative with a descriptive design. Nine primary care centres were included in the intervention project and four in the control group. The interviewees in the reported sub-study were selected from five municipalities i.e., representing all the primary care centres included in the intervention.

### Participants

A total of 1600 older persons were included; 800 were included in the intervention and 800 in the control group. From the intervention group, 20 older persons were selected for the reported sub-study. Semi-structured interviews were conducted individually with the 20 selected older persons. The interviewees consisted of eight women and 12 men, aged 76–93 years who were living in three small or middle-sized municipalities and two larger cities in the Region of Östergötland, Sweden.

A purposive sampling strategy were used (Curtis et al., 2000). All of the participants were recruited from the patient list from the primary care centres that were included in the FPC project. The responsible nurses at each of the primary care centres recruited the older persons from the patient lists. The selection criterion for the nurses was that the research team wanted names from four persons over 75 years (two men and two women). A time selection principle (Creswell & Poth, 2016) was also used in order to pick out patients; the research team asked the nurses to provide contact details of the four first patients that matched their criteria and that were treated at the primary care centres during the month of November 2017. When being interviewed the selected patients had been included in the intervention for ten months.

### Data collection and access

After receiving the contact details from the nurses, the interviewing researchers sent an information letter about the sub-study to the older persons and contacted them by telephone asking if they were willing to participate in an interview. In all, 37 older persons were approached and 20 agreed to take part in an interview. Seventeen (six men and 11 women) declined to participate. The persons who declined to take part did so due to health-related issues or because they did not wish to be interviewed. The data was collected from December 2017 until July 2018. Three of the researchers (AO, EC and AS) conducted the interviews which took place in the homes of the older persons. The interviews lasted between 27 minutes to one hour and 54 minutes each. Throughout three of the interviews the older person’s spouses was present for part of the time during the interview. The spouse’s accounts were transcribed as a part of the conversation but not analysed. A semi-structured interview guide was used (can be sent on request). After an opening presentation of the project the interview focused on the participants experiences around three main areas namely “everyday life/care”, “autonomy/own impact on health care/services” and “the future”. In this article the analysis is concentrated mainly on answers concerning the first two themes. The interviews were audio-recorded and transcribed verbatim by a professional transcriber. However, the interview extracts presented in the results section have been edited and repetitive talk has been removed. Places where part of a statement or the following sentence has been removed are marked with (…).

### Ethics

Confidentiality for the participating older persons was ensured at all stages of the research. The older persons gave their written consent to take part in the sub-study. The overall intervention project and our sub-study had both received ethical approval from the regional Ethics Committee in XXX in Sweden.

## Analysis

The analysis in this sub-study was based on segments from the interviews that concerned the participants’ views on participation in their own care and treatment. Content analysis, inspired by Graneheim et al. (2017) was used in the analysis. In the sub-study, we have used the International Classification of Functioning, Disability and Health (ICF) definition of participation as *Participation is involvement in a life situation* (World Health Organization [WHO], 2001) as it provides a scientific basis for understanding the concept. In relation to the analysis we have extended it to include “focusing on how and to what extent the older person themselves defined their involvement in care” as such, participation is considered relative to all contextual factors concerning health situations. Intrinsically, in our analysis we have used this definition as a starting point when going through the material focusing on what opportunities and limitations the older persons see for participation and individual solutions in the care provided.

In the first step of the analysis four of the authors (AO, EC, EV and AS) conducted a naive reading of all the transcribed interviews, sorting out sections that related to experiences of care. After the first sorting process the research assistant focused on searching for meaning units which consisted of talk associated with participation in relation to the ICF definition and put those statements into a table. Thereafter, a condensation of all the statements concerning participation was made by the first author (AO) and the research assistant (EV). Meaning units related to the same content were grouped together and condensed into codes by the first author. The codes were then sorted into subcategories and those were condensed into categories by the first author. The first author had the main responsibility for performing the different steps in the analysis from dividing and condensing the meaning units into codes and thereafter into subcategories and categories. To increase the credibility the subcategories and categories were checked by the other authors. A discussion took place after an independent coding process undertaken by all authors. To establish reliability the text was read individually by three of the authors (AO, EC and AS). For consistency, the authors discussed the coding of units of meaning, subcategories and categories until consensus was reached. By means of this process, a set of categories were derived which could characterize the perceptions of participation in care of frail older persons with significant care needs.

## Results

In almost all the 20 interviews both opportunities and limitations were mentioned by the older persons when experiences of participation in care were talked about. Four categories were revealed:

- *Involvement on organizational levels*
- *Conditions for taking part in care*
- *Involvement in direct care or treatment*
- *Views of receiving care in relation to autonomy*

Beneath each of the categories were subcategories. Both categories and 12 subcategories are illustrated in Table I below.LE

**Table I.** Categories and subcategories (divided into opportunities and limitations for participation)

Categories	Subcategories and examples in the study	
	Opportunities	Limitations
Involvement on organizational levels		Shortcomings in organization between different health care organizations Poor coordination between different professions
Conditions for taking part in care	Professional treatment, Continuity of staff Possibilities to influence care	Unprofessional treatment Large staff turnover Must orientate through the health care system themselves
Involvement in direct care or treatment	Content with treatment and arrangements of care Trust in health care professionals Satisfied with own opportunities obtaining desired services	Discontent with treatment and arrangements of care Lack of trust in health care professionals Difficulties obtaining desired services Requests for other types of care/services than those offered
Views of receiving care in relation to autonomy	Independence- can choose when care and services are inserted Self-determination—monitor their situation, how care should be performed	Reduced independence Negative to be dependent on help Cannot control the planning of care and services

In the presentation of the results beneath, each category will be exemplified with quotes that illustrates how experiences were expressed in relation to participation in the care by the older persons. The first names of the older persons in the quotes are all fictitious names given by the researchers.

### Involvement on organizational levels

The category involvement on organizational levels includes the subcategories, *Shortcomings in organization between different health care organizations and Poor coordination between different professions*. I this category it was noted that experiences reported about opportunities for participation on a structural level i.e., between different health care organizations and different stakeholders, were few. Also, statements from the interviewees concerning this category were only found when it came to reflections in relation to negative experiences of participation. Those limitations were statements that evolved around shortcomings between different health care organizations. Below is a quote from the subcategory *shortcomings in organization between different health care organizations* where a woman in her talk compares her experiences between treatments at the breast unit at the hospital and her contacts with the primary care centre. In her account she describes her positive experiences at the hospital of having a nurse that had a coordinating function, “someone who holds all threads”.

*Norah:* *And that’s the key contact person I think you should have. And that’s the contact I want to get at the primary care centre. The contact in … well I’ve had breast cancer two more times and had that key contact person. And that has been wonderful. (Interview 14, Norah 80 years)*

The interviewee cited above stated that the treatment at the hospital was very good since she had a responsible nurse who took a holistic perspective towards her care, but also towards other practical things around her such as communicating with her relatives and contacting the municipality care manager and the occupational therapist when going home for assistance with home care and assistive technology etc. In the interview she also requested that this type of coordination function should be incorporated between organizations and also be made available at the primary care centre. These kinds of statements about requests for more structure in the organization of care, for example, to have someone at the primary care centre that is responsible for the coordination of all health care contacts, were quite common (they occurred in seven of 20 interviews) in the material. Also, inadequacies in coordination between health care organizations and staff were highlighted in the interviews. The next quote also illustrates the subcategory *shortcomings in organization between different health care organizations* highlighting the experienced lack of synchronization between acute hospitalized care and primary care. In the quote, a woman is talking about her various contacts with different health care organizations when she had a heart attack:

*Bridget:* *Then three doctors came … different ones. (p. 9) … Yes. But all of them, they said … how did you get here? Well but I took a cab, I said. Did you take a cab? Yes, I said. And then the chief physician came and he said … no, you need to get to XXX town right away … over to XXX town. Oh? I said then. And … but then when I was at … in XXX town, and they had done this widening (of vessels), then one says … I was to be transferred to YYY town again then, then that professor comes and then he says like, why are you nagging so much about … because I thought, like … I told the nurses … are they not talking to each other? What is this? I said. Should three (doctors) … what are they doing? (p. 10) (Interview 2, Bridget 88 years)*

In the episode the woman is describing a situation where she experienced a lack of coordination between different health care facilities within the same organization and between the nurses and doctors in respective facilities. She had to meet three different doctors and she was transferred between different care facilities (both hospitals and a primary care centre). Bridget is questioning whether the staff cannot communicate better with each other. Like Bridget several of the interviewed older persons in the study mentioned that they wanted that health care staff in primary care centres should work on improving the communication between different health care providers (primary care, hospitals and community care). These kinds of statements on an organizational and structural level were almost exclusively around issues of a lack of coordination between different organizers of health care and different professions. Also noted in our material was that these comments were only mentioned in relation to the interviewee’s negative experiences of participation.

### Conditions for taking part in care

The category Conditions for taking part in care contains the subcategories, *Professional/unprofessional treatment, Continuity of staff/large staff turnover, Possibilities to influence care, Must orientate through the health care system themselves*. This category was concerned more with the circumstances for being involved on an organizational and interpersonal level and how the older persons could be involved in the care and treatment. The subcategories include themes such as whether or not there was continuity in relations with staff and whether the treatment was professional. An issue for the interviewees was whether they had opportunities or felt limited in relation to influencing their care and treatment. Furthermore, an issue that diminished their participation, and that was common in the material, was communication difficulties with health care staff. The following quote is from the subcategory *possibilities to influence care* and illustrates a man answering a question about how he experiences possibilities to present his views on the suggestions of treatment provided by health care professionals:

*Stephan:* *You can say that. And I don’t think I’ve ever come across that they say “no, no, it’s not like that”. They take it in. Or maybe they are so smart that they keep a straight face and let me carry on and come up with my ideas.*

*In:* *Mm. Right. So it’s not that they contradict you, but they listen?*

*Stephan:* *No, I haven’t experienced that actually. (Interview 19, Stephan 78 years)*

The man highlights that he feels that he can present his thoughts and opinions about how he thinks the care around him should be performed and organized. He also expresses some insecurity about whether he gets recognition for his views and opinions from health care staff. We interpret this as him wishing for more confirmation of his requests and suggestions. Thus he concludes that he finds all health care staff that he meets to be empathetic and inclusive towards him and his needs. This was a common opinion among almost all interviewees. Absence of coordination between different care providers and to not be included on an interpersonal level in the contacts with different health care staff could also be regarded as a limitation in relation to participation. The following quote from the subcategory *must orientate through the health care system themselves* where a woman illustrates how she chose to handle her experienced shortage of participation in the care provided by different stakeholders:

*Norah:* *There was no one holding the whole thing together. And then I think we wrote a letter (…) that the primary care centre should be the one that keeps the whole thing together then. And that they send for help if needed but the whole overall responsibility should be there. And then I think, after that it has become better (…) So that … but it was my own initiative to write.*

*…*

*Norah:* *Well, I was going there and then I had it with me and read it out loud.*

*…*

*Norah:* *And then it became like more of this change then as well that it was that she … it might have come later, but … yes a year later. Because she is working on it and thinking about how it should be and that it should not be too many, but it should be the one who …*

*…*

*Norah:* *And the nurse is also familiar with how I am and such. So in that way it feels safer now with the primary care centre. … (Interview 14, Norah 80 years)*

Norah, cited above, claims that she did not feel that she was in control of her healthcare contacts, which led to her taking care of it on her own with the help of relatives. She contacted the primary care centre as they were the responsible organizers for overall care. She then stresses that she got positive attention from the responsible nurse, after seeing the letter, where the primary care centre after this episode took more general responsibility for her care situation, except that it was still difficult to get in contact with the primary care centre by phone. These types of statements were common in our material. Several of the interviewees highlighted that they often themselves had to guide themselves through the health care system that was often perceived as difficult to understand and manoeuvre and this limited the experience of participation.

### Involvement in direct care or treatment

The category involvement in direct care or treatment included the subcategories, *Content/discontent with treatment and arrangements of care, Trust/ lack of trust in health care professionals, Satisfied with own opportunities obtaining desired services’, Difficulties obtaining desired services, Requests for other types of care/services than those offered*. This category concerned experiences that were related to participation in the direct care/treatment and whether the older persons had the opportunity to participate in and influence how care and treatment should be performed. Also, the possibilities, or lack of them, for self- managing the planning of care were highlighted. Trust in the health care staff was consistently a clear sub-category in this category. In our sub-study, overall the interviewees wished to retain a long-lasting relationship with the primary care centres and other care providers such as hospitals and community-based home care. The older persons with significant care needs saw stability and continuity in the health care contacts they had as important factors in relation to possibilities for participation. The following quote is an example of how a woman acts to maintain stability in her contacts with primary care. The reported episode is from the subcategory *content with treatment and arrangements of care* where she is talking about when she moved and was asked a- question from her GP about transferring her health care contacts to another primary care centre closer to her new home:

*Sara:* *No … I thought it was so good at the time. Bosse (the GP) then asked me when I moved … do you want to stay here? he said. Do you want to remain at XXX primary care centre? I will not go to the YYY primary care centre, I will stay here, I said. Then he just laughed at me. So that is good . Instead of sitting there and rattling off everything for a new one then. No? (Interview 18, Sara 74 years)*

The continuity in health care contacts seemed to be interwoven with the older person’s contentment with care as well as their own ability to influence care. This is highlighted in the quote above when the woman stresses that one of her reasons for continuing being a patient at her primary care centre is the possibility to have a continuous relationship with the same GP and that she does not have to describe all her conditions to a new GP. In the material these were issues highlighted by all the interviewees as important factors for creating a sense of participation. Similar arguments could be found in relation to limitations in the involvement in care where the older persons experienced difficulties in getting care and services as desired. There were also requirements for other types of examinations/treatments than what could be offered that limited their sense of participation. Below, is an example from the subcategory *difficulties obtaining desired services* where a man called George expresses his concerns about his experienced limitations in relation to his own participation:

*George:* *… maybe it was a year or so, I just thought I’d take one of these PSA tests. But it was not possible (…).*

*I:* *Yes. Because you wanted to take it for preventive purposes?*

*George:* *Yes, that’s right. In case … yes. But in any case then I called again and said that I had a hard time and got up a lot at night and, so I got an appointment there. But around the same time I got that blood clot in ‘2016 then. And then they took it. So I never went to take that test. And then the primary care centre, I also find odd (…) they do not check your cholesterol level. And I would think they should really check it because … the diet may very well affect that I get these blood clots. I think. (Interview 7, George 84 years)*

In the example above this man says that he experiences a lack of influence when it comes to decisions about tests regarding his symptoms. He expresses a wish that the nurse at the primary care centre had been more sensitive to his needs. Although he called and pointed out the need for a test he experienced that the test fell between the cracks when other symptoms appeared that needed treatment.^1^ In this example, the man perceives that he has rights to demand a certain treatment that he thinks is needed. This episode could be seen as characteristic in our material in terms of expressing exclusions from decisions relating to tests, which led to patients’ dissatisfaction with the participation in their own care situation.

### Views of receiving care in relation to autonomy

The category Views of receiving care in relation to autonomy includes the subcategories, *Independence- can choose when care and services are inserted /reduced independence, Self-determination—monitor their situation, how care should be performed, Negative to be dependent on help, Cannot control the planning of care and services*.^2^ These statements revolved around whether or not they could choose when and where care and services were provided. Self -determination was highlighted in relation to monitoring and controlling the care situation. To be dependent on care and not being able to influence its execution were described in some interviews as representing an intrusion in personal autonomy. In the quote below, from the subcategory *self-determination—monitor their situation, how care should be performed* where a man is describing the strategy he used to ensure that he had control as he actively monitored his care situation:

*Daniel:* *I have the same nurse that I meet. There has been some rotation among them but I become friends with them … my doctor then, she has the nurse as an intermediary … And then maybe she tells me something and then the doctor calls me. So sometimes I think … am I prioritized or what the hell is that? You start to wonder. Because I think I get attention that I cannot imagine that … But maybe it depends on the person too, because after all I’m … monitoring and can speak up and have some ideas and opinions about how it shall be done and sometimes I might tell doctors what to look at and what it is that’s … yes. So maybe it depends a little on the person as well. (Interview 4, Daniel 87 years)*

The man Daniel cited above stresses in the example that he is keen to create a relationship and become friends with the staff at the primary care centre. He further elaborates that he feels privileged that he is prioritized in his care contacts and he is thinking about whether it may be because of his personal characteristics, i.e., he is able to speak up and give his opinion. This can be seen as a way of creating self-determination in the sense that he can monitor his overall care situation in regard to how care or treatment should be executed, resulting in maintained independence. Furthermore, a lack of autonomy in relation to participation in care were also visible in the material. The following quote is from the subcategory *negative towards being dependent on help* were a woman is talking about needing care in the home, and it illustrates the importance of independence even when living with multiple health problems:

*Bridget:* *No, I don’t do that. But I’m aware that when I have to … first of all my daughter who is in the archipelago … says mother, if you would like help with … then I can prepare food for you then. No, but I do not want that right now, but as long as I can … otherwise you will be … you won’t think you’ll succeed with anything. (Interview, 2 Bridget 88 years)*

Bridget states that she will only accept care and support if it is necessary. She wants to cope herself as long as she can and with as few services as possible. Her daughter offers help with food when needed, which Bridget has declined for now. She stresses the importance of autonomy and that receiving care can result in that you are thinking that you cannot succeed with anything. The woman’s statement exemplifies a common aspect in the material, namely a fear that being dependent and losing control leads to reduced self- determination. For many of the participants in our sub-study, reduced independence was seen as something negative in relation to participation. It was stressed that being dependent and lacking control over the planning of care and services was mainly regarded as negative. Although the interviewees in our research had significant care needs, autonomy and independence emerged as important factors that were closely linked to the older persons’ experiences of participation.

## Discussion

The main finding from this sub-study was that although the interviewed frail older persons had significant care needs, all of them stated a strong willingness to participate in and have control over the care provided by different health and primary care organizations. This shared image is consistent with other studies that have shown that older persons want to be involved in care (e.g., Baastiens, 2007, Sixsmith et al., 2014). Our analysis adds to this research, as these views seem to be constant, even though the older persons had significant care needs living at home. Furthermore, following the intervention project Focused Primary Care (FPC) it was clear that the older persons experienced that, as regards the logic of the care system (health and primary care) and its ways of allocating services, both had opportunities and limitations in regards to their abilities to participate in care. The interviewed older persons did not discuss participation in relation to primary care so much given that the intervention was based in a primary care context. This could be a result of our sub-study being conducted early in the intervention and they had not yet experienced the potential differences between traditional Primary care where there is usually no specialized elderly team. Another reason for this could be that different primary care centres could have had varying abilities to communicate to the older persons that they were participants. It might also be a consequence of how successfully different primary care centres were at implementing the intervention Focused Primary Care (FPC), in regards to the formation of the “elderly team” depending on local and personnel factors.

However, at a concrete level we found that the older people interviewed, in general, experienced both opportunities and limitations in relation to participation on an interpersonal level (meso- and micro levels). Nevertheless, they experienced only a lack of/ limitations in relation to possibilities of participation in care on organizational levels. It was highlighted that it was difficult to understand the organizational system underpinning care, with the result that many were left to orientate themselves through the system, which limited their experience of participation.

In relation to individual conditions for taking part in care in the present study, the results showed that overall, the older persons experienced high satisfaction with their own possibilities to express their views on and be directly involved in care and treatment. However, what sometimes limited their sense of participation were difficulties experienced in getting care and services as desired or when they had requirements for other forms of care then what could be offered within the organization. These results agree with previous research (c.f. Breitholtz et al., 2012, Doyle et al., 2012) where negative experiences of care are often linked to limitations within the care organization, whereas the relational dimensions of caregiving are emphasized as the most central (positive) aspects of caregiving for older persons (G. Harvey et al., 2018; Lambotte et al., 2020). Our sub-study clearly shows that interpersonal relations with staff were of great importance regarding whether or not frail older persons with significant care needs experienced possibilities for participation in the execution and performance of their care.

The results also show that participation limitations were concerned the older person’s experiences of shortcomings in relation to influencing the organization of care in and between different health care organizations. It can be difficult to comment on the contribution of the intervention in terms of concrete impacts of the older person’s experiences on an organizational level. This result can be discussed in relation to findings from other studies that highlight that patient participation sometimes is reduced to an interpersonal level (Ceci & Purkis, 2009) were more basic accounts of patient choice and autonomy is considered as good enough involvement in care from the perspective of health and care organizations (Mol, 2008; Sinding et al., 2012). In the present study it is difficult to say if this was the case. Our result however indicates that the older person’s difficulties to understand the organizational underpinning system can be a result of a patient discourse were older persons are supposed to take a greater responsibility for their own care (Foster et al., 2017; McDonald et al., 2007) which includes being an active agent in navigating the these systems. This is further a debate that intersects with the pressures to decrease costs for health care also with the intent on limiting older peoples dependence on recourses and services (see for example, Harvey et al., 2018; Meyer et al., 2018)

However, what we can say, was that many of the older persons gave similar accounts and quite a few claimed that they felt selected and/or prioritized in their contacts with the health and primary care services. Several persons also highlighted the fact that they saw primary care as functioning as a coordinator for finding solutions to their individual care needs. This suggests that the older persons might have had some awareness of the organizational intentions of the intervention and/or the care system that they were a part of. This also further highlights the role of the elderly team and the coordinator at the primary care centre, which had an important function in this intervention. Good personal relations and continuity in these relations are important factors affecting older persons’ ability to express their needs to someone who also could understand and influence their care. Further research has also shown that the organization of care between different stakeholders often restricts older people’s scope for autonomy (Fjordside & Morville, 2016; Sundström et al., 2018), which in some respects can be a result of the hierarchal structure of the care organization that is characterized by bureaucracy. However, this could also be linked to the attitudes imbued in Western cultures where high values of independence (e.g., Lawrence, 2017) can influence the older person’s willingness to seek help and to be an active agent in making demands on information exchange and on the content and scope of care. This may perhaps also to some extent explain the expressed contentment that was expressed by the older persons in terms of opportunities to influence care. In summary, the results suggest that participation described by frail older persons with significant care needs seems to be interwoven with a desire for an understanding of the structure of the care system on an overarching level as well as on an interpersonal and personal level. To have agency and control over the care situation in our sub-study seemed to require a balance among influence, self-determination, and intrusion of personal autonomy. Participation in care, for the interviewed older persons, thereby addressed both physical and emotional aspects as well as informed information of the care system to receive supported independence, and by extension an experience of genuine involvement.

## Limitations

Although some parts of the results are encouraging, we want to discuss the limitations of the sub-study. Firstly, the design of the research where the sample of participants was selected from a time selection principle. In retrospect, one might ask if the older persons could have had clearer opinions on participation if the selection of participants had occurred at a later stage when the intervention had been ongoing longer, after approximately two years. Therefore, there is an incentive to conduct a follow-up study to determine if this is the case. The research procedure that was used also resulted in quite many older persons declining participation in the sub-study (n 17). The older persons who agreed were probably motivated to be interviewed and had views and opinions about the care provided and their possibilities to influence it. There were also an over- representation of men among the interviewed older persons. We cannot know the possible influence on the results from this selection bias through this small sample. However, similar statements from both men and women were found in all of the categories and sub-categories suggesting that this over- representation was not an important factor for the results. Also, the interviews were different regarding length. This could have been because there were three of us conducting the interviews and we had different attitudes as individual interviewers towards letting the older person speak freely and pose follow-up questions. It could moreover be a consequence of the older persons’ different health statuses, and their conversational abilities and their willingness to speak in detail about their living conditions. Finally, we used qualitative content analysis and the results cannot be generalized to all the adults included and should be interpreted with caution. Although themes were identified in the interviews, they were based on individual accounts of the participating older person’s experiences. The strengths of the research approach lie in its ability to provide descriptions of the way in which people understand their own reality, as the approach involves an interpretive analysis of the underlying meaning of the data.

## Conclusion

The results of the reported sub-study indicate that there is a need for fewer actors in health and primary care and better coordination between organizations in regards to sharing information. Further care is a complex phenomenon that has several layers that need to be taken into consideration to facilitate participation and supported independence for frail older persons. The results from our analysis show that frail older persons with significant care needs share challenges with lack of clarity in the organizational structure of care. They find having transparency on an organizational and structural level as helpful. Our argument is therefore that participation in care should not be reduced to an interpersonal level but also include organizational and structural levels and this could be improved further when designing interventions. Our sub-study furthermore clearly highlights that an ongoing relationship with one care stakeholder with an overall responsibility is important for providing care for frail older persons living at home. Also, continuity of staff and having a specialized elderly team were ongoing relationships with the same staff members is regarded as positive. To keep care contacts together a coordinating person is needed who safeguards the relationship with the older person. There is considerable potential for developing the health and primary care sector to better target the needs of frail older persons with significant care needs and enhance their participation and independence. Interventions, like the one followed in this project, can play a critical role in realizing the needs of older persons, where providing participation in care is recognized as a significant goal in order to assist them to manoeuvre the care puzzle.

## Notes

1. Worth noticing here is that the Swedish Health and Medical Care Law (2017, p. 30) states that individuals do not have the right to demand health care treatment and/or tests. As an individual you have the right to have a number of treatments offered and explained, and then you have the right to choose between them.

2. The category contains some overlaps with the subcategory *Continuity of Staff* in the category *Conditions for taking part in care*, but the content is more focused around personal concerns about the interviewee’s view of receiving care, and care in relation to autonomy and independence.
